# *Empedobacter brevis* Meningitis in a Neonate: A Very Rare Case of Neonatal Meningitis and Literature Review

**DOI:** 10.1155/2016/7609602

**Published:** 2016-08-25

**Authors:** Deepak Sharma, Ankur Patel, Priyanka Soni, Pradeep Sharma, Basudev Gupta

**Affiliations:** ^1^NEOCLINIC, Everest Vihar, TN Mishra Marg, Nirman Nagar, Jaipur, Rajasthan, India; ^2^Setu Newborn Care Centre, Ahmedabad, Gujarat, India; ^3^Department of Microbiology, JLN Medical College, Ajmer, Rajasthan, India; ^4^Department of Medicine, Mahatma Gandhi Medical College, Jaipur, Rajasthan, India; ^5^Department of Pediatrics, Civil Hospital, Palwal, Haryana, India

## Abstract

*Empedobacter brevis* is gram-negative bacilli that belongs to Flavobacteriaceae family. It was previously known with name of* Flavobacterium breve*. The reservoir of these bacteria is soil, plants, water, food, hospital water sources, including incubators, sinks, faucets, tap water, hemodialysis systems, saline solutions, and other pharmaceutical solutions. We report a case of term female newborn, admitted with complaint of respiratory distress developing soon after birth and developed clinical features of sepsis at age of 92 hours of postnatal life. The sepsis screen was positive and blood culture and cerebrospinal fluid showed growth of* Empedobacter brevis* that was resistant to multiple antibiotics. The neonate was treated with appropriate antibiotics and was discharged successfully. The novelty of the case report is that this is the first case report of neonatal sepsis caused by *Empedobacter brevis*.

## 1. Introduction

*Empedobacter brevis* previously known with name of* Flavobacterium breve* belongs to Flavobacteriaceae family. It is an environmental, gram-negative bacteria and is rarely encountered in human specimens. The pathogenicity of the bacteria is rare and is usually limited to health care workers [1]. We report first case of neonatal bacteremia and meningitis secondary to* Empedobacter brevis*. All the previous six case reports have been published in adults with various manifestation. The neonate was treated with appropriate antibiotics and was discharged.

## 2. Case Presentation

A term female newborn (40 weeks) with birth weight being 3200 grams (appropriate for gestational age) was admitted to the neonatal intensive care at three hours of postnatal life with complaint of respiratory distress developing soon after birth. The newborn was born to G3P2L2A0 mother by elective cesarean section, indication of elective cesarean section being previous cesarean section. The neonate had respiratory distress and hence was started on hood box oxygen. The neonate was evaluated with chest radiograph, which had features of transient tachypnea of newborn. The neonate was started on minimal feeds and intravenous fluid. There was gradual improvement in respiratory distress in the next 24 hours but neonate still had requirement of oxygen. Tube feeds were given to the baby and were gradually increased. At age of 92 hours of postnatal life, the neonate developed features of sepsis in the form of decreased activity, cool and dusky periphery, off colour, pale, delayed perfusion (capillary refill time more than 4 seconds), feed intolerance, abdominal distension, feeble pulses, tachycardia (170–180 beats/min), low blood pressure, and new onset of respiratory distress. The neonate was evaluated with sepsis screen, blood culture, and chest radiograph and was started on antibiotics (cefotaxime and amikacin) in suspicion of late onset sepsis (LOS). The neonate required multiple vasopressors and steroids for septic shock. The neonatal sepsis screen was suggestive of leucocyte count of 2400/microliter (leucopenia), platelet count of 40000/microliter (severe thrombocytopenia), Immature neutrophil/Total neutrophil (I/T) ratio of 0.42, toxic granulations on peripheral smear, and very high C-reactive protein (22 mg/dL) (normal value less than 1 mg/dL). The infant was given supportive care with packed cell transfusion for anemia and platelet transfusion of severe thrombocytopenia. Chest radiograph was suggestive of left middle and lower zone pneumonia. Lumbar puncture done in view of LOS was suggestive of meningitis (cerebrospinal fluid (CSF) sugar being 31 mg/dL against simultaneous blood sugar of 104 mg/dL, CSF protein being 206 mg/dL, and cell count of 45 cells/microliter with 85% cells being neutrophil). The blood culture that was sent from two different sites using strict aseptic precaution using three swab techniques showed growth of* Empedobacter brevis*. The organism was sensitive to tetracycline, tigecycline, and cefoperazone-sulbactam and was resistant to ciprofloxacin, colistin, amikacin, amoxyclav, ampicillin, cefazolin, cefepime, cefotaxime, ceftazidime, cefixime, ertapenem, gentamicin, imipenem, levofloxacin, meropenem, piperacillin + tazobactam, piperacillin, and tobramycin. The CSF culture also showed* Empedobacter brevis* growth with similar sensitivity pattern. The antibiotics were upgraded to intravenous cefoperazone-sulbactam as per the sensitivity pattern. Gradually the neonate improved and vasopressors were tapered and stopped. The blood culture and CSF examination was repeated on day 14 of cefoperazone-sulbactam that was suggestive of no bacterial growth and CSF showed normal study. The intravenous antibiotics were given for 21 days as neonate had CSF culture growth. The neonate was monitored with weekly head circumference growth that was normal and head ultrasound showed no ventricular dilation or features of ventriculitis. The infant was discharged on breast feeding in well condition with no neurological deficit. Surveillance culture was sent to trace the source of infection but we were not able to trace the source of infection in the index case. The infant was evaluated with MRI brain in follow-up which was suggestive of normal study and there was no developmental delay in the infant.

## 3. Discussion 

*Empedobacter brevis* was previously known as* Flavobacterium breve* and is a gram-negative bacillus and belongs to the Flavobacteriaceae family. It is an environmental inhabitant occasionally encountered in human specimens. These organisms can be found in various niches. Most significant in clinical relevance is their ability to survive in hospital environment, especially in moist areas of the hospital [1, 2]. Because of their ability of survival in hospital areas, these organisms have the potential to contaminate laboratory culture media and blood culture systems. Whenever these bacteria are encountered, their clinical significance and potential for contamination should be seriously considered. The reservoir of these organism is soil, plants, water, food, hospital water sources, including incubators, sinks, faucets, tap water, hemodialysis systems, saline solutions, and other pharmaceutical solutions [3–5]. The mode of transmission that has been postulated is exposure of patients to contaminated medical devices or solutions, but source is usually not always known [6].

As environmental organisms, no specific virulence factors have been identified for these species. However, the ability to survive in chlorinated tap water may give these organisms an edge in their ability to survive in hospital water systems. The development of infection basically requires exposure of debilitated patients to a contaminated source. The bacteremia is often associated with implanted devices, such as catheters, ventriculostomy catheters, or contaminated medical solutions [6].

Gram staining is used to detect these organisms in clinical material.* Empedobacter brevis* varies in being short to long nonmotile rods. Most strains grow at 37°C and all strains grow at 30°C temperature. They are obligate aerobes and form circular, smooth, shiny with entire edge, light yellow colony on 5% sheep blood agar. They are nonlactose fermenters on MacConkey agar [1, 2]. The key biochemical characteristic has been summarized in Table 1.

There are no definite treatment guidelines as there is lack of validated in vitro susceptibility testing methods for these bacteria. In general, these species are frequently resistant to beta-lactams and aminoglycosides that are commonly used to treat infections caused by gram-negative bacilli. An unusual feature of many of these species is that they often appear susceptible to and are treated with antimicrobial agents usually considered effective against gram-positive bacteria like clindamycin, rifampicin, and vancomycin. There are no recommended vaccination or prophylaxis protocols for these bacteria because of their ubiquitous nature and generally it is not considered. Hospital acquired infections can be controlled through the use of appropriate sterile technique with implementation of strict protocols for sterilization and disinfection of hospital supplies [1, 6].

A search of literature of previous published case report showed only six reports of* E. brevis* infections; all were in the adults and none in neonates.

Schroeder et al. reported case of 60-year-old male, who developed septic shock and multiorgan dysfunction secondary to accidental intravenous infusion of a nonsterile saline solution. The blood culture showed growth of multi-drug-resistant* Empedobacter brevis*. The isolate was only sensitive to ciprofloxacin, imipenem, trimethoprim/sulfamethoxazole, and tetracycline. The patient was discharged after treatment with ciprofloxacin. This case report highlighted the importance of use of sterile saline solution in hospitals [3].

Bokhari et al. reported case of* E. brevis* bacteremia in a 69-year-old male patient with HIV infection. The patient was admitted with black tarry stools, malaise, nausea, and vomiting. The blood culture sent on days 1, 2, and 3 of admission showed growth of* E. brevis*. The organism was sensitive to fluoroquinolones, trimethoprim-sulfamethoxazole, tigecycline, polymyxin-B, and piperacillin-tazobactam. The patient was treated with piperacillin-tazobactam and was discharged [7].

Janknecht et al. reported a case series of* Empedobacter brevis* endophthalmitis after cataract extraction. In this case series, twelve patients were referred because of endophthalmitis after 1–6 days of uncomplicated cataract extraction. The mean age of the patients was 75 years.* Empedobacter brevis* was found in the anterior chamber and in the vitreous of eleven subjects. The patients were treated with intravitreal antibiotics initially with vancomycin and amikacin and later as per sensitivity pattern of culture.* E. brevis* was sensitive to ampicillin, piperacillin, imipenem, and quinolones. Inadequate sterilization process was considered as the possible cause of this epidemic outbreak [8].

Nishio reported case of 83-year-old woman with anaphylactoid purpura, redness, blisters, and erosion of foot.* Empedobacter brevis* was cultured from right feet lesion and was sensitive to minocycline hydrochloride. Local application was started with minocycline hydrochloride and the woman showed improvement [9].

Chi et al. in their retrospective study of 197 ventriculostomy drains placed in 155 patients reported 28 infections out of the 197 (14.2%) drains. The mean age of the cohort was 60.8 years. They found one infection of* Empedobacter brevis* in this cohort [6].

Raman et al. reported a 65-year-old female who developed right knee cellulitis secondary to* Empedobacter brevis* infection. The blood culture showed growth of* Empedobacter brevis* which was sensitive to the majority of antibiotics. The patient was treated with levofloxacin and was discharged in well condition [10].

The novelty of the index case is that this is the first case of* Empedobacter brevis* sepsis in neonatal period that leads to bacteremia and meningitis. The infant responded well to cefoperazone-sulbactam and patient was discharged in well condition. The blood culture and CSF culture before discharge showed no growth.

## 4. Conclusion

*Empedobacter brevis*, also known as* Flavobacterium breve*, is a gram-negative bacillus and belongs to the Flavobacteriaceae family. It is an environmental inhabitant and is occasionally encountered in human specimens. There are only few case reports of* Empedobacter brevis* causing infection in human being, and our case report is the first of its type. Its multidrug resistant nature makes it a serious problem for treatment of* Empedobacter brevis* sepsis and health care workers should follow strict asepsis precaution to prevent its transmission to patient.

## Figures and Tables

**Table 1:** Table showing various biochemical characteristics of *Empedobacter brevis*.

Biochemical characteristics	Results
Mannitol	Negative
Indole	Positive
Gelatin	Positive
Urea	Negative
Nitrate reduction	Negative
Esculin hydrolysis	Negative

## Abbreviations

CSF: Cerebrospinal fluid
I/T ratio: Immature neutrophil/Total neutrophil ratio
LOS: Late onset sepsis.
